# Performance of α‑, β- and γ‑GeSe Monolayers for Near-Field Radiative Heat Transfer: An Ab Initio Study

**DOI:** 10.1021/acsomega.5c09056

**Published:** 2025-11-13

**Authors:** André Gusso, Francisco Sánchez-Ochoa, Raúl Esquivel-Sirvent

**Affiliations:** † Departamento de Ciências Exatas-EEIMVR, 28110 Universidade Federal Fluminense, Volta Redonda 27255-125, Brazil; ‡ Instituto de Física, 7180 Universidad Nacional Autónoma de México, Ciudad de Mexico 01000, Mexico

## Abstract

In this work, the performance of three polymorphs of monolayer GeSe for near-field radiative heat transfer (NFRHT) applications is compared. The α, β, and γ phases of this interesting and novel two-dimensional (2D) semiconductor have been extensively investigated in many aspects and are promising materials for applications in NFRHT. While the performance of the α and β phases has already been investigated separately in the literature, no consistent and systematic comparison between the performance of the three phases has been performed. For a consistent comparison, the relevant physical parameters, like the effective electron masses and optical conductivities, were calculated using density functional theory. To increase the heat flux, the monolayers are assumed to be n-doped. It was concluded that despite some striking differences in their optical conductivities, which can vary from isotropic to highly anisotropic, the three phases can deliver similar maximum heat fluxes. This finding contradicts what is usually expected: that highly anisotropic 2D materials that can sustain hyperbolic plasmon-polaritons can be expected to deliver larger heat transfers compared to nonhyperbolic materials. This result evidence that other physical properties of real materials can be more relevant for optimizing the NFRHT.

## Graphical abstract

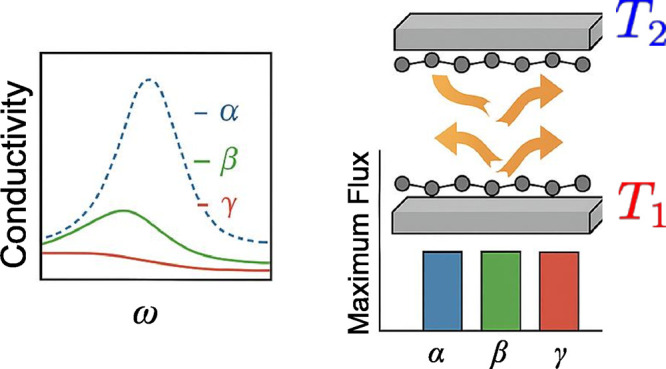

## Introduction

Radiative heat transfer refers to thermal energy transfer through electromagnetic waves, specifically through the emission, absorption, and scattering of photons.^1,2^ The thermal emission depends on the distance from the body up to the Wien thermal wavelength, after which it follows the Stefan–Boltzmann law and is no longer dependent on the distance, as has been verified by a number of experiments.^3–6^ The so-called near-field radiative heat transfer (NFRHT) occurs because of the evanescent electromagnetic fields close to the surface.^7^ Due to the evanescent electromagnetic waves, the heat flux exceeds that predicted by Stefan-Boltzmann’s law.^8^ The main features that determine the radiative heat transfer between bodies are their emissivity related to the temperature gradients and the dielectric properties of the materials.^9^

NFRHT has potential applications in energy management, heating and cooling control at the nanoscale, and the design of thermal transistors^10^ and rectifiers.^11^ The heat transfer control depends on the dielectric properties of the material and the ability to excite surface-polaritons.^12^ This has opened the door to search for suitable materials ranging from simple polaritonic materials,^13–15^ and metasurfaces^16,17^ as well as Dirac materials,^18^ topological,^19,20^ and Weyl type materials.^21^

The particular physical properties of 2D materials have also attracted attention for their possible use in NFRHT.^22,23^ Graphene has been widely studied for that purpose.^24,25^ It can, for instance, be used to suppress heat transfer by rotating two plates at magic angles,^24,26,27^ or as buffers on structured surfaces. Heat radiation between graphene and hBN^28,29^ has been shown to be useful by combining the surface response of each material, as well as graphene and hexagonal boron nitride (hBN) structures.^30^ Furthermore, the NFRHT for 2D materials is expected to be different from that for bulk materials because of the difference in the optical properties of the materials and the boundary conditions imposed upon the optical modes. An intermediate regime is possible with layered systems made of 2D materials.^31,32^

Recently, we studied the NFRHT effects between β-GeSe monolayers using a combination of density functional theory (DFT) and Rytov’s theory of fluctuation electrodynamics.^33^ Monolayer GeSe was chosen since it was shown previously that its α phase yielded a heat transfer larger than that predicted for graphene.^34^ For the β phase of GeSe, the large anisotropy was expected to boost the large radiation process. However, a direct comparison between the results for the α and β phases obtained in these different works was not possible because of the very distinct approaches used to calculate the optical properties of the monolayers.

Studying the NFRHT between the different phases of GeSe monolayers would be important for several reasons, particularly within the context of nanophotonics, thermoelectric devices, and 2D material physics, where fine-tuning of the band structures and properties is important. In this work, we are going to study theoretically and compare the NFRHT behavior between the α, β, and γ phases of GeSe monolayers. These three phases have different electronic band structures and phononic modes, which can affect how each of the phases emits or absorbs thermal radiation. Understanding NFRHT across different phases allows for the phase-engineered control of heat flow at the nanoscale. Comparing the performance of the three phases is also an opportunity to check whether the large optical anisotropy of the β-GeSe results in the expected enhanced heat flux due to hyperbolic plasmon-polaritons.

## Methods

### GeSe Phases and System Configuration

The three phases of GeSe have different physical properties that are worth understanding to check their effect on the NFRHT.^35–38^ In Figure 1a–c, we show the three GeSe allotropes as monolayers with different 2D Bravais lattices, orthorhombic or hexagonal lattice. The side views in Figure 1d–f show the layer thickness and the different atomic arrangement in the **a**-**c** plane. Once a phase is selected, to calculate the NFRHT, we consider two parallel monolayers separated by a distance *d*. This separation is kept much larger than the lattice constant *a* so that the monolayers can be treated as a 2D optical system. Each layer is at different temperatures, *T*_1_ and *T*_2_, where *T*_1_ > *T*_2_. This is shown in Figure 1g.

**Figure 1:**
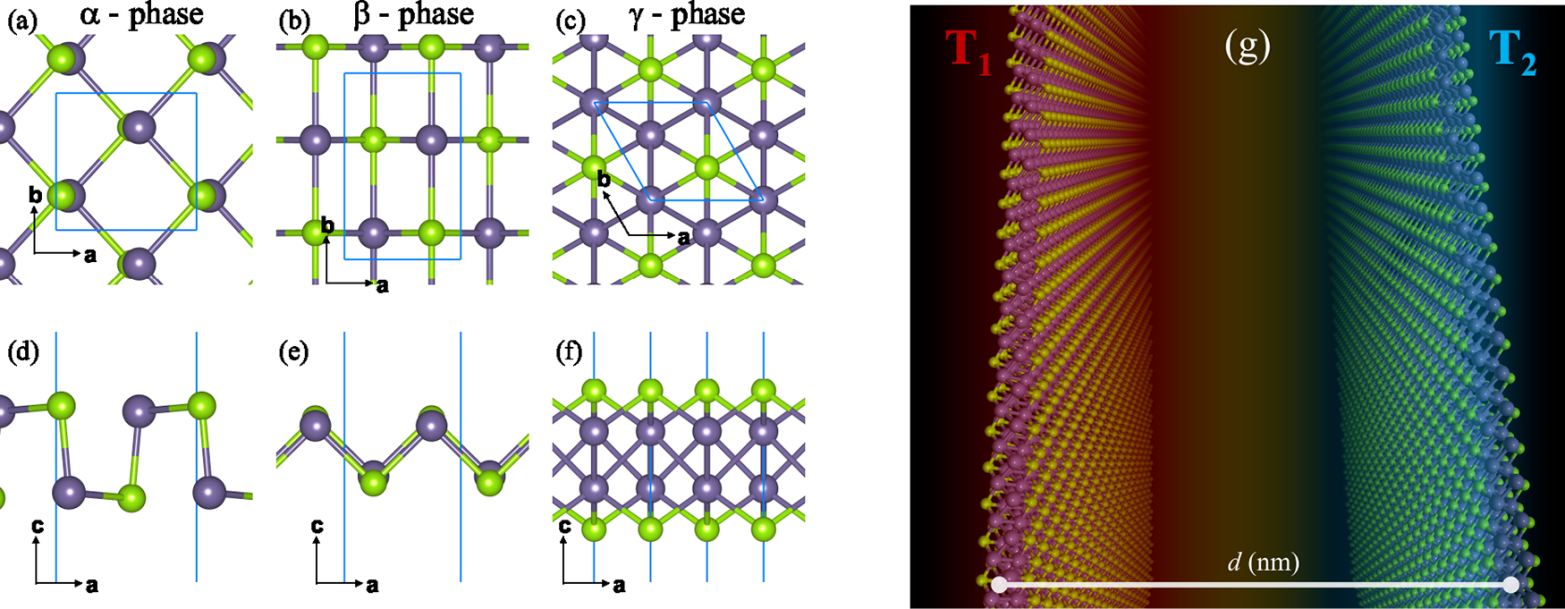
(a–c) top and (d–f) side views for the atomic models of α, β and γ phases of GeSe. The Ge and Se atoms are colored gray and green, respectively. The blue line delimits the unit cell. (g) two monolayers of γ phase separated by an interlayer distance several orders greater than its lattice constant, *d* (nm) > *a* (Å).

### Near-Field Radiative Heat Transfer

In this section, we briefly introduce the theory for calculating the near-field radiative heat transfer. According to Rytov’s theory of thermally stimulated electromagnetic fields, the radiative heat transfer has a contribution of propagating electromagnetic fields (far field) and evanescent fields (near field).^7,8^ The near-field is mainly determined by the evanescent modes in the gap between the two surfaces, when *d* < λ_T_, with λ_T_ being the Wien thermal wavelength.^39^ The main idea is that at any nonzero temperature, microscopic charge and polarization fluctuations exist inside dissipative media. These random sources radiate electromagnetic fields. Rytov’s theory models those sources statistically by solving Maxwell’s equations using a Green’s function formalism to obtain the local density of states between the bodies.^40,41^ The density of states that couples both surfaces can be described by the photon transmission coefficient $\xi (\omega ,d,\vec{k})$ given by

$$\xi (\omega ,d,\vec{k})=\{\begin{array}{l} \mathrm{Tr}[(I-R_{2}^{†}R_{2})D^{12}(I-R_{1}R_{1}^{†})D^{12†}],\;k_{\left|\right|}<\omega /c\\ \mathrm{Tr}[(R_{2}^{†}-R_{2})D^{12}(R_{1}-R_{1}^{†})D^{12†}]e^{-(2|k_{z}|d)},\;k_{\left|\right|}>\omega /c \end{array}$$ 1

In this equation, $k_{\left|\right|}=\sqrt{k_{x}^{2}+k_{y}^{2}}$ denotes the magnitude of the component of the wavevector $\vec{k}$ parallel to the 2D sheets, which lie in the *x*–*y* plane, while $k_{z}=\sqrt{k_{0}^{2}-k_{\left|\right|}^{2}}$ , corresponds to its perpendicular component if the wavevector in vacuum is *k*_0_ = ω/*c*. I = diag­(1, 1) and **R**_*j*_ (*j* = 1, 2) is the reflection matrix of *s* and *p*–polarized waves

$$R_{j}=\left[\begin{array}{ll} r_{j}^{s,s} & r_{j}^{s,p}\\ r_{j}^{p,s} & r_{j}^{p,p} \end{array}\right]$$ 2

The components *r*_*j*_^α,β^ are the reflection for an incident wave of polarization α that is reflected in a polarization β. In terms of this reflection matrix, we define **D** ^12^ = (I – **R**_1_ **R**_2_ *e* ^–(2*ik*_*z*_ *d*)^)^−1^. In eq 1, the first case corresponds to the propagating or far-field modes, and the second one to the near-field modes due to evanescent waves. While, in principle, the contribution of the transmission coefficient of the propagating modes should also be included in the calculation of the PTC, their contribution is negligible at the short separations we consider in our analysis,^42^ and this correction becomes unnecessary. The expressions for the reflection coefficients for an anisotropic layer are given in refs 43,44 . The optical properties of each slab are needed to calculate the reflectivity matrix and NFRHT, which we are going to obtain using DFT.

The total heat flux (THF) between two layers is calculated using the relation^23,45^

$$Q=\int_{0}^{\infty }q(\omega )d\omega =\frac{1}{(2\pi {)}^{3}}\int_{0}^{\infty }\left[\int_{-\infty }^{\infty }\int_{-\infty }^{\infty }\xi (\omega ,d,\vec{k})k_{\left|\right|}dk_{\left|\right|}\right]\times [\Theta (\omega ,T_{1})-\Theta (\omega ,T_{2})]d\omega$$ 3

where *q*(ω) denotes the spectral heat flux (SHF) and the function Θ­(ω,*T*) corresponds to the mean thermal photon energy at frequency ω and temperature *T* (Planck function),

$$\Theta \left(\omega ,T\right)=\frac{\hbar \omega }{\left[\exp\left(\hbar \omega /k_{B}T\right)-1\right]}$$ 4

where *k*_B_ is the Boltzmann constant.

### Computational DFT Methodology

To calculate the band structure, effective masses, and the optical properties of the different GeSe phases, we performed the numerical calculations with the Vienna ab initio Simulation Package (VASP)­code.^46^ The projector-augmented wave (PAW) method^47^ was used to describe the core–valence electron interactions. Since the highly anisotropic atomic and electronic structures of GeSe phases are very sensitive to exchange-correlation approximations,^33,36,37,48–50^ here we adopt the Perdew–Burke–Ernzerhof (PBE) parametrization,^51^ within the generalized gradient approximation (GGA) for solids, termed as PBEsol,^52^ to describe the electronic exchange-correlation energy as in previous reports.^33,53^ The lattice vectors and atomic positions were fully optimized, with a threshold value of 0.01 eV for residual forces, using an energy cutoff of 450 eV. Because the GeSe monolayer can adopt different Bravais lattices, the sampling of reciprocal spaces was set to Γ-centered 10 × 10 × 1, 16 × 8 × 1, and 10 × 10 × 1, within the Monkhorst–Pack scheme,^54^ for the α, β, and γ phases, respectively. A Gaussian smearing of 0.01 eV was used for Fermi surface broadening. For electronic self-consistency, a 10^–8^ eV value was set. The supercell method was employed to simulate the monolayers with a vacuum space of 15 Å to avoid spurious interactions between replicas. To get the optical properties of the different phases of the GeSe homostructures we resort to ab initio calculations within the framework of DFT as in our previous works^33,53^ to determine de linear optical properties such as the frequency-dependent complex dielectric function, ε_3D_(ω), after that the corresponding bulk optical conductivity, σ_3D_(ω) = *i*[1 – ε_3D_(ω)]­ε_0_ω, and finally the 2D optical conductivity, σ_2D_(ω) = *L*σ_3D_(ω), being *L* the slab thickness. We employ a Lorentzian broadening of 75 meV for the optical calculations.

This 2D conductivity includes only the contribution of the interband transitions, and while it is more pronounced in a range of photon energies above that of the conduction gap, it can also be sufficiently large in the infrared region to contribute to the NFRHT. To take advantage of the heat flux enhancement usually obtained due to the plasmon-polaritons, we consider that the GeSe monolayers are doped with negative carriers (electrons). In this case, the total surface conductivity tensor receives two contributions σ̅ = σ̅^inter^ + σ̅^ep^, where the first term comes from the DFT calculation (interband transitions), and the last one is the contribution from free electron plasma and is calculated using the Drude model as

$${\overset{-}{\sigma }}^{\mathrm{ep}}=\frac{in_{e}e^{2}}{m_{e,\kappa }}\frac{1}{\omega +i\gamma_{e}}$$ 5

where *n*_e_ is the electronic density, and *m*_e,κ_ the effective masses along κ = (*x*, *y*) and γ_e_, the electronic damping. Changing the values of *n*_e_ changes the overall conductivity tensor.

It is worth noting that in our previous studies^33,53^ we have included the contribution of the optical phonons to the conductivity of β-GeSe. In the present work, we ignore this contribution. The main reason is that the optical phonons are relevant to the NFRHT for small *n*_e_, and in this work, we consider comparatively large values of *n*_e_, because it helps to increase the heat flux.

## Results and Discussion

### DFT Calculations

The structural, energetic stability, and electronic information obtained with the total energy DFT formalism of different GeSe phases are summarized in Table 1. Since there are two pairs of Ge and Se atoms in each cell, we compare the total energies for the three phases to determine the relative energetic stability among the systems. The relative energy reported in Table 1 shows that the most stable GeSe monolayer is the γ, then α, and last the β phase. Note that the asymmetry in the lattice vectors for the β phase promotes high in-plane anisotropy in its electric and thermal conductivity. The β phase is obtained under high pressure, resulting in a distorted α phase.^38^ The γ phase adopts a hexagonal structure,^55^ and depending on strain, the electronic configuration can exhibit topological or semimetallic behavior.

**Table 1:** General Properties of Monolayers of Different GeSe Phases^a^

phase	structure	*a*, *b*	RE	*E*_g_ (T)
α	O	4.05, 3.95	311	0.83 (I)
β	O	3.58, 5.73	523	1.44 (I)
γ	H	3.75, 3.75	0	0.54 (I)

a Structure with orthorhombic (O) or hexagonal (H) symmetry; *a* and *b* lattice constants in Å; Relative energy (RE) in meV/f.u., where f.u. means formula unit; fundamental band gap (*E*_g_) in eV plus the type (T) of the transition: direct (D) or indirect (I).

Figure 2 shows the electronic band structures calculated along the high symmetry points of the irreducible Brillouin zone (see the right panels). Notice that, while the electronic structure of α and γ phases has a nearly isotropic behavior around the Γ point for the last occupied band and first unoccupied band, the β phase shows an anisotropic character around the same Γ point but solely for the electrons. The three GeSe phases present indirect electronic band gaps with values less than 1.5 eV within the current approximation of exchange and correlation functional (see Table 1). The electronic anisotropies in the α and β phases can be observed in the calculated effective electron masses along the perpendicular directions around the Γ point, as seen in Table 2. It is worth mentioning that the γ phase has the lighter negative carriers with average values of *m*_e_^*^ ∼ 0.167*m*_0_. The β-phase reaches the lowest (largest) value of effective mass along the Γ → X (Γ → Y).

**Table 2:** Effective Electron Masses, *m*_e_^*^, in Units of *m*_0_, Calculated along Perpendicular Paths around the Γ and A Points^a^

phase	*m*_e_^*^
α	0.125 (A → B), 0.186 (A → Y)
α′	3.454 (Γ → X), 3.591 (Γ → Y)
β	0.149 (Γ → X), 6.218 (Γ → Y)
β′	2.455 (A → B), 0.085 (A → Y)
γ	0.166 (Γ → K), 0.169 (Γ → M)

a *m*_0_ is the bare electron mass. Notice that α and α′ are not different phases, but the effective masses are calculated along different high-symmetry points. The same applies to β and β′.

**Figure 2:**
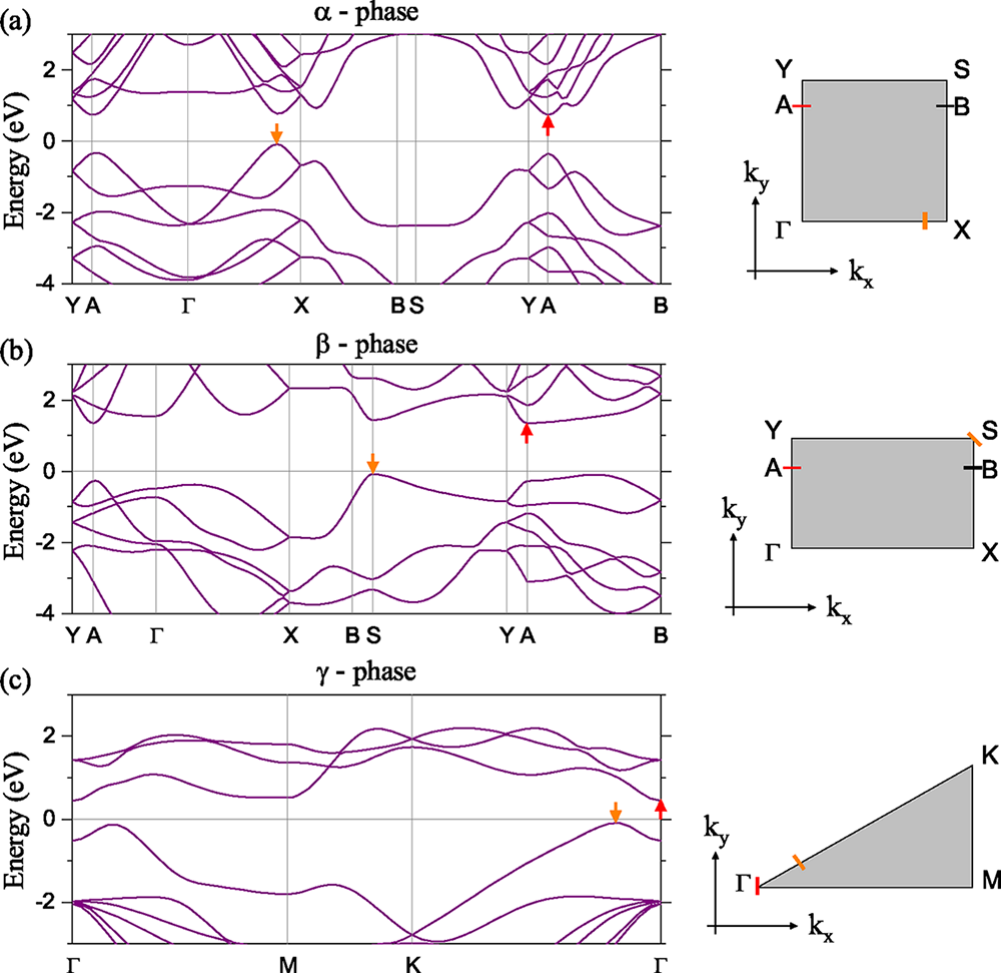
Electronic band structure calculated along the path connecting the high symmetry points depicted in the irreducible Brillouin zone in gray color. (a) α-GeSe, (b) β-GeSe, and (c) γ-GeSe. In all panels the Fermi energy is set to 0 eV. Orange arrows denote the maximum of the valence band, while the orange thicks denote the corresponding position along the high-symmetry lines in the irreducible Brillouin zone.

Additionally, we calculated two other sets of effective masses around the A point, where the global minimum of the conduction band in Figure 2 occurs (as indicated by red arrows in the band structure). This additional point of high symmetry exists for the α and β′ phases. See Table 2. This is because an in-plane uniaxial mechanical tensile strain of 2% applied on the β phase, along the zigzag direction (see Figure 1c), can switch the conduction band minima from A to Γ in Figure 2b.^48^

The results from DFT for the 2D conductivity due to the direct interband electronic contributions to the GeSe conductivity are presented in Figure 3, for the three GeSe phases and along the *X* and *Y* directions. For the α-phase, the real (a) and imaginary parts (b) show a small anisotropy. For the β-phase, both the real (c) and imaginary (d) parts exhibit strong anisotropy. Finally, the γ-phase conductivity due to the interband transition is completely isotropic.

**Figure 3:**
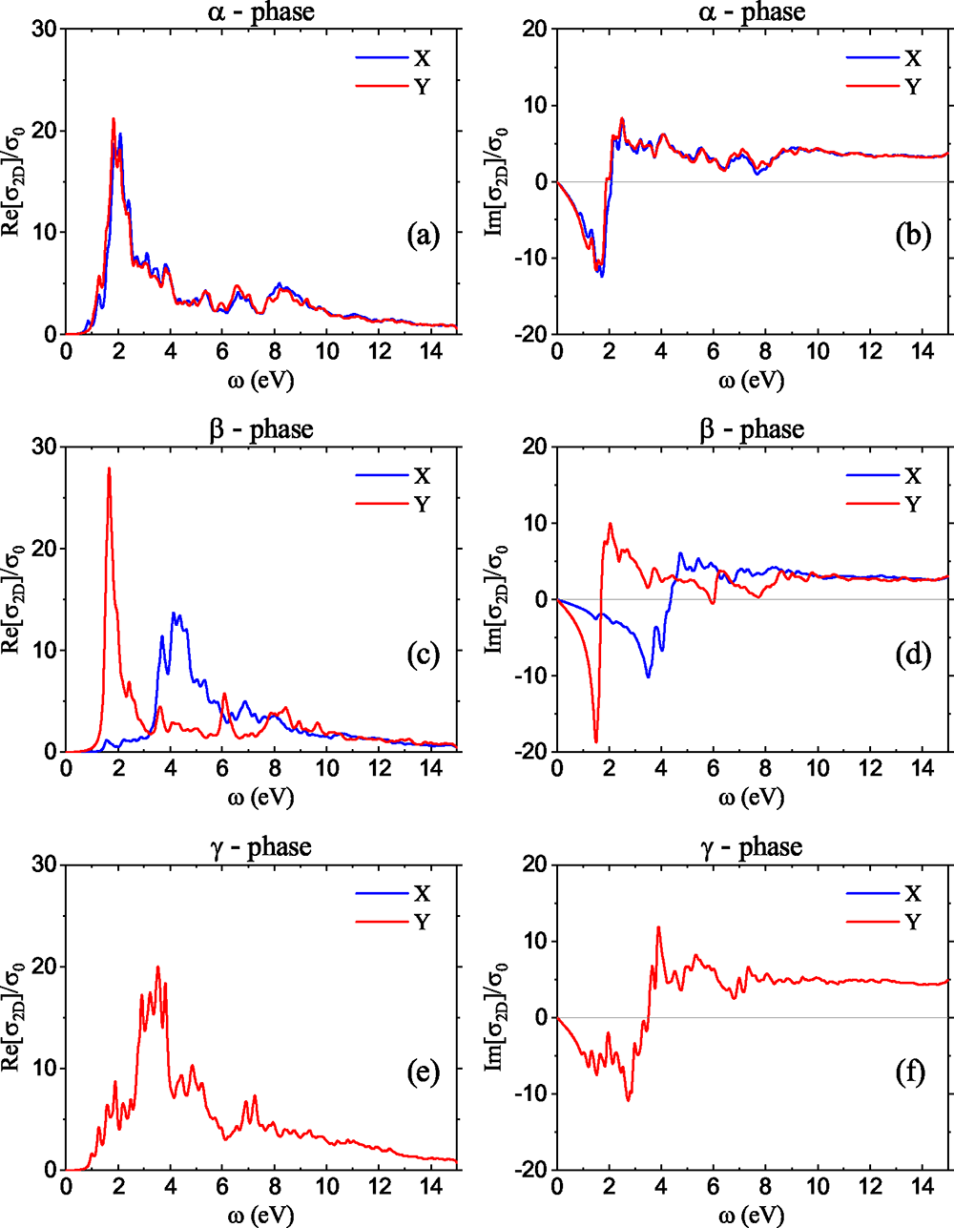
Real (Re) and imaginary (Im) part of two-dimensional optical conductivity due to the electronic interband transitions, σ_2D_, calculated along two perpendicular directions, *X* and *Y*, for three different phases of monolayer GeSe: (a,b) α, (c,d) β and (e,f) γ phase. The conductivities are normalized to σ_0_ = *e* ^2^π/2*h*.

### Radiative Heat Transfer

The more relevant parameter that characterizes the performance of a given material or device concerning NFRHT is the total heat flux *Q*. Here, we assume a particular temperature difference between the two 2D sheets, namely, *T*_1_ = 400 K and *T*_2_ = 300 K in the calculation of *Q*. Considering higher temperatures *T*_2_ could require us to take into account the changes of physical parameters of the GeSe monolayer with the temperature, which we expect to be sufficiently small between 300 and 400 K that they can be ignored.

In Figure 4, the *Q* predicted for the three GeSe phases is presented as a function of *n*_e_ and *d* on a log scale. The results for α′ are not shown because this phase performs quite poorly, delivering much lower *Q* values. This is attributed to the very large effective electron masses, which require very large electron densities to result in a plasma frequency in the spectral region that is relevant for the heat transfer.

**Figure 4:**
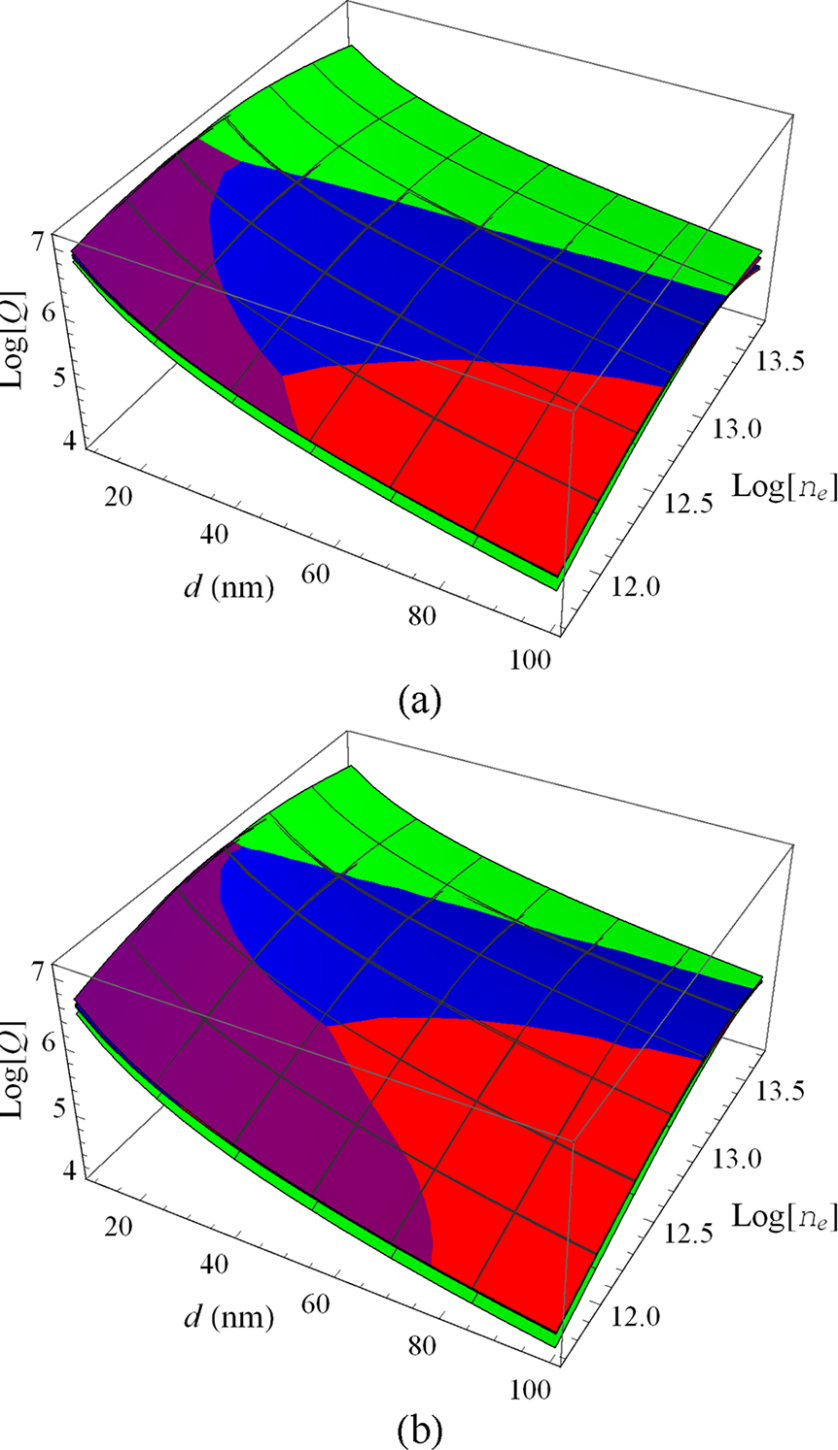
Log-scale plot of the total heat flux (*Q*) between monolayers of the α (red), β (green), β′ (purple), and γ (blue) GeSe phases as a function of the log of *n*_e_ and the separation *d*. In (a) γ_e_ = 0.01 eV while in (b) γ_e_ = 0.03 eV. The electron density *n*_e_ is varying between 0.5 and 50 × 10^12^ cm^–2^.

Figure 4 shows that each phase can prevail in different regions in the *n*_e_ × *d* plane. These regions are also going to depend upon other parameters, like γ_e_, as it is illustrated by the differences between Figure 4a,b, obtained for γ_e_ = 0.01 and 0.03 eV, respectively.

A more detailed comparison of the performance of NFRHT for the different GeSe phases can be done based on the curves for *Q* × *n*_e_ for different separations shown in Figure 5. From both Figures 4 and 5, it is possible to see that the maxima of *Q* for the α, γ, and β′ phases occur at similar values of *n*_e_ at a given separation. For the β phase the maximum *Q* is obtained for significantly higher values of *n*_e_. It is important to highlight that these maxima correspond to heat fluxes that are about 12 × 10^3^ times the flux between black bodies at the same temperatures *T*_1_ = 400 K and *T*_2_ = 300 K for *d* = 10 nm. This heat flux is several times larger than that predicted from ab initio calculations between graphene sheets^42^ for the same temperatures and separation.

**Figure 5:**
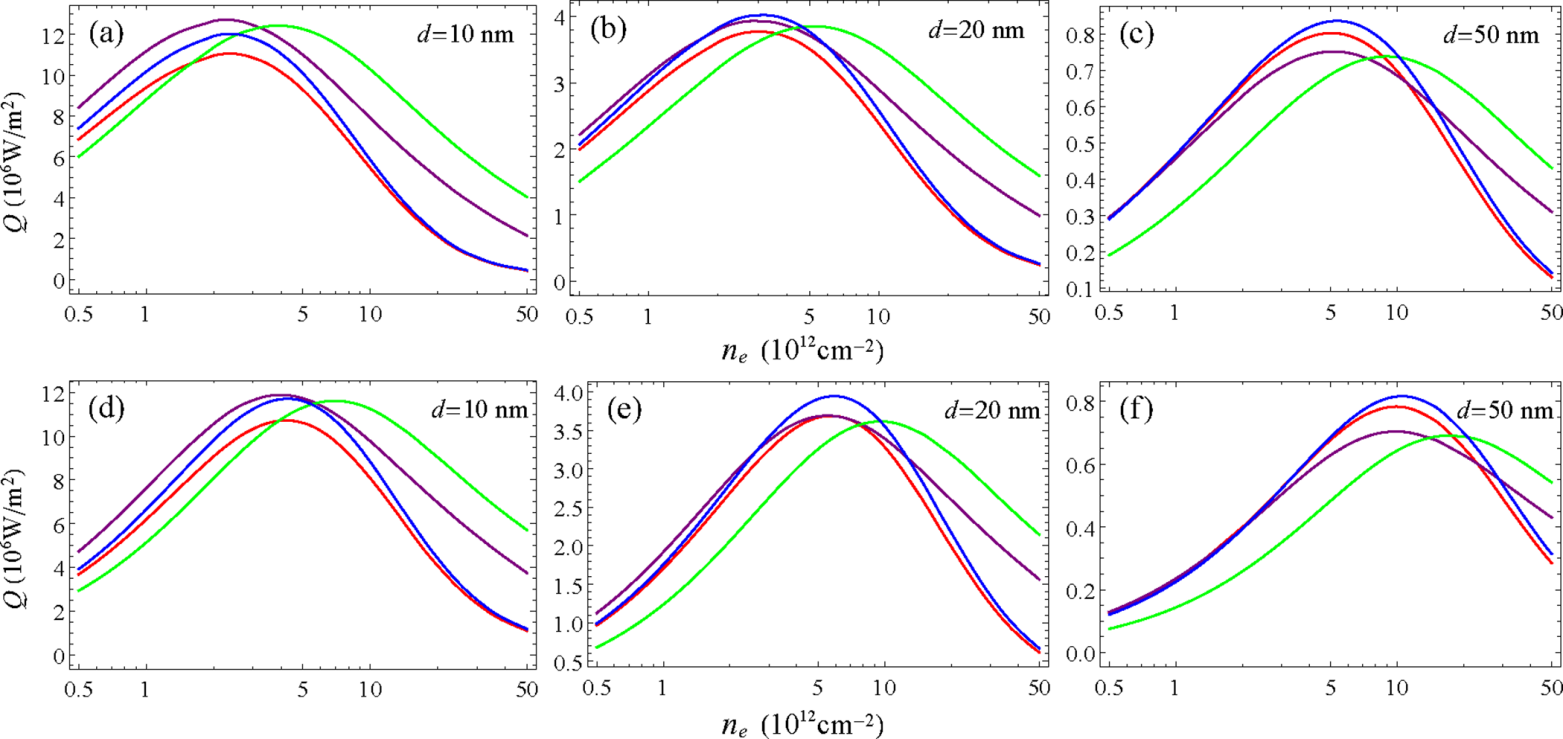
Total heat flux (*Q*) between monolayers of the α (red), β (green), β′ (purple), and γ (blue) phases as a function of *n*_e_ for (a) and (d) *d* = 10 nm, (b) and (e) 20 nm and (c) and (f) and 50 nm. Results for γ_e_ = 0.01 eV (upper panels) and γ_e_ = 0.03 eV (lower panels).

More specifically, from Figure 5, we see that for γ_e_ = 0.01 eV, the maximum *Q* is attained for *n*_e_ around 2 × 10^12^ cm^–2^ at *d* = 10 nm and increases to 5 × 10^12^ cm^–2^ at *d* = 50 nm for the α, γ, and β′ phases. For the β phase, the maximum is found at 4 and 10 × 10^12^ cm^–2^ for the same range of separations. As already noted in previous works by the authors^33,53^ the maximum of *Q* is going to be displaced to higher values of *n*_e_ if γ_e_ increases. It was found that *n*_e_ for the maximum *Q* almost doubles when γ_e_ changes from 0.01 to 0.03 eV as can be seen in Figure 5.

While the value γ_e_ = 0.01 eV is representative of a very low and optimistic relaxation frequency, and 0.03 eV is a more realistic value, γ_e_ is not known a priori and is costly to evaluate from first-principles analysis. While it can hardly be expected to be below 0.01 eV, γ_e_ could be larger than 0.03 eV. Since this parameter can vary significantly, we have further analyzed how the maximum *Q* varies with γ_e_. In Figure 6a, we present a representative graph of *Q* × *n*_e_ for the β phase and a separation *d* = 10 nm. Similar curves were obtained for the other phases and separations. We can see that as γ_e_ varies from 0.01 to 0.06 eV, the *n*_e_ for the maximum *Q* increases steadily. For the phase being considered, it changes from *n*_e_ ∼ 3 × 10^12^ cm^–2^ for γ_e_ = 0.01 eV up to ∼10 × 10^12^ cm^–2^ for γ_e_ = 0.06. This change by an approximate factor of 3 was also observed for other phases. These large differences in *n*_e_ for the maxima demonstrate the importance of a correct estimate of γ_e_ to adequately predict the NFRHT effects. If, for instance, γ_e_ is assumed to be 0.01 eV and in an actual GeSe monolayer it results to be 0.06 eV, then if the separation is *d* = 10 nm, *n*_e_ would have been set to 4 × 10^12^ cm^–2^. Based on the curves in Figure 6a, the attained heat flux would be half of the expected one.

**Figure 6:**
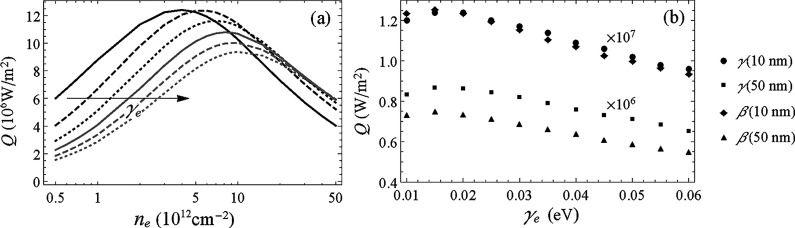
(a) The total heat flux (*Q*) between monolayers of the β phase as a function of *n*_e_ for *d* = 10 nm. (b) Maximum attainable *Q* as a function of γ_e_ for the β and γ phases at representative gaps of 10 and 50 nm.

To further understand how the maximum *Q* depends on the relaxation frequency, we show in Figure 6b *Q* as a function of γ_e_ for the γ and β phases (similar results were found for the α phase). This figure highlights that for γ_e_ between 0.01 and 0.03 eV, the maximum *Q* does not vary appreciably. However, as γ_e_ increases, the maximum attainable flux decreases steadily, being about 25% smaller for γ_e_ = 0.06 eV in all cases portrayed in Figure 6b.

Concerning the maximum attainable heat flux *Q* for the three different phases, they present a similar performance at all separations, with the differences not exceeding 20%. Considering the uncertainties inherent to the DFT calculations of the optical properties and effective masses, it is possible to conclude that the different phases exhibit similar performances. This is an unexpected result, considering that we are comparing very different crystalline arrangements, which, as we have seen in previous sections, have quite different properties, such as gaps, effective masses, optical responses in the interband region, and levels of anisotropy. Later in this section, we discuss the reasons for the similar performances of the α and γ phases. However, the fact that the highly anisotropic β phase has a performance close to that of the other phases remains an outstanding result. Because the β-GeSe monolayer presents large frequency ranges with the properties of a hyperbolic material,^53^ it could be expected to deliver larger heat fluxes,^42,56^ compared to the other phases.

Going beyond the analysis of the maximum *Q*, Figure 5 reveals that the curves for *Q* × *n*_e_ for the α and γ phases are quite similar, being essentially the same or very close for all the different separations. Nonetheless, α-GeSe tends to slightly outperform the γ phase for *d* up to 50 nm. The tendency of the curves for *Q* of the α and γ phases to get closer as *d* increases was observed up to the largest separation of 100 nm that we have investigated. Now, compared to the β phase, the α- and γ-GeSe deliver significantly higher values of *Q* at lower doping levels, being surpassed only by the β′ phase at short separations. In its turn, for larger *n*_e_, the β phase dominates. This last result can be seen as a consequence of the significant displacement of the curve of β-GeSe to higher *n*_e_.

The observed differences and similarities between the curves for *Q* for each phase can be attributed to the interplay between the contributions to the total optical conductivity coming from the free electron plasma and the tail of the imaginary part of the conductivity due to the interband transitions in the infrared region. The contribution of the free electron plasma is determined by the effective electron masses and damping γ_e_. The tail of the interband transitions is going to be defined by the gap and the intensity of the main peak of these transitions. The 2D conductivity resulting from the sum of these two contributions is illustrated in Figure 7, where the vertical blue line separates, approximately, the region with lower frequencies, where the free electron plasma dominates σ_2D_, from that of higher frequencies, where the tail of the interband transitions is more important. The frequency range that contributes the most to the final *Q* is going to depend upon *d* and *n*_e_. At large separations or small *n*_e_ lower frequencies are more relevant, and the properties of the free electron plasma are going to determine *Q*. At short separations *d* and large *n*_e_, higher frequency values are more relevant, and the interband transitions play a more significant role in determining *Q*. Based on these general observations, it is possible to further understand the heat flux obtained for the different phases.

**Figure 7:**
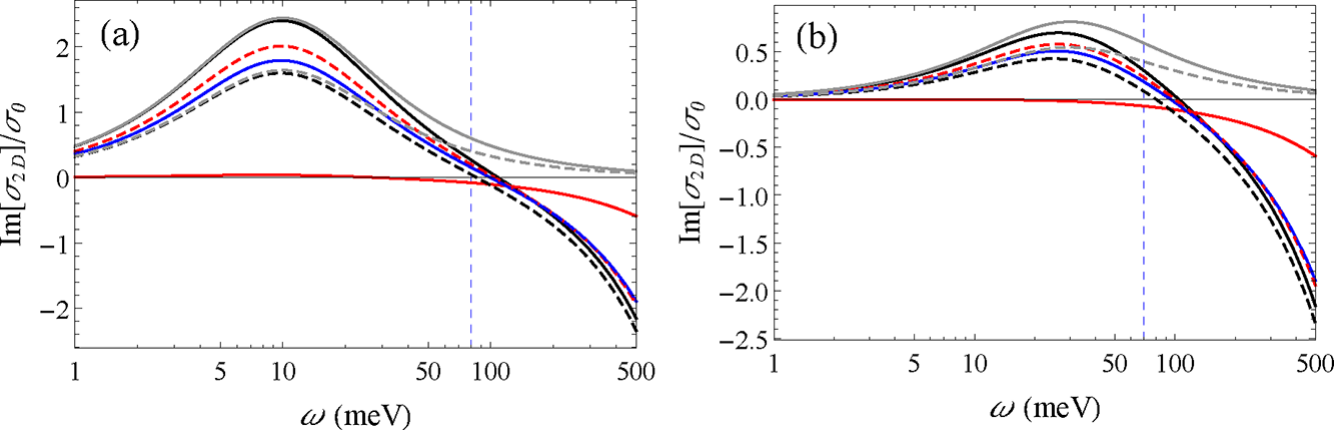
Imaginary part of the complex conductivity (normalized by σ_0_) as a function of angular frequency ω calculated assuming *n*_e_ = 2 × 10^12^ cm^–2^ and (a) γ_e_ = 0.01 eV and (b) 0.03 eV. The continuous­(dashed) lines represent the conductivities along the *x*(*y*) directions. The α-, β- and γ-GeSe correspond to the black, red, and blue curves, respectively. For comparison, the gray curves correspond to the conductivity of the α phase without the contribution of interband transitions. The vertical blue line separates, approximately, the regions of low and high frequencies in which the contributions of the free electron plasma and the interband transitions dominate the conductivity, respectively.

The contribution of the free electron plasma is defined by *m*_*x*_ and *m*_*y*_, which are different for the three phases. While the effective electron masses for the α and γ phases are close, there are significant differences in the optical conductivities along the *x* and *y* directions at low frequencies, as can be seen in Figure 7. This could lead us to expect more significant differences in the predicted *Q* for the two phases. However, the actual dependence of the heat flux on the optical properties that are defined by *m*_*x*_ and *m*_*y*_ is such that these two phases end up delivering similar values of *Q*. The dependence of the heat flux on the effective electron masses can be obtained by analyzing the expression for $\xi (\omega ,d,\vec{k})$ in eq 3 calculated assuming that σ_*x*_ and σ_*y*_ are given only by the Drude plasma conductivity, eq 5. It results that $\xi (\omega ,d,\vec{k})$ is a function of the effective masses combined in two terms of the form *M*_±_ = *m*_*x*_^–1^ ± *m*_*y*_^–1^. These two combinations have approximately the same value for the α and γ phases: *M*_+_ = 13 and *M*_–_ = 2.6, and *M*_+_ = 12 and *M*_–_ = 0, respectively. This explains the very similar value of *Q* for these two phases at large *d*, where the contribution of modes with lower frequencies dominates and the optical properties are defined by the free electron plasma. The *Q* for β′ is also close to those of these two phases because *M*_+_ = 12 also has a similar value. The differences come from the fact that while for the α and γ phases *M*_–_ is small or exactly zero, we have *M*_–_ ∼ 11 for β′. In its turn, for the β phase *M*_+_ ∼ *M*_–_ ∼ 7, differing significantly from the values for all the other phases and leading to the discrepancy in the curves for *Q* at large *d*.

For small separations *d* < 20 nm, *Q* for α- and γ-GeSe are still very close but differ slightly more. This result is explained by the fact that the optical properties of these two phases at higher frequencies are quite similar. To observe this, we assume that the interband transitions are dominated by a single Lorentz oscillator. In this case, the imaginary part of the conductivity in the near-infrared and the reststrahlen region is well approximated by

Im[σ]=−ϵ0(ϵ∞−1)ω=cω 6

where ϵ_0_ is the vacuum permittivity and ϵ_*∞*_ is the high-frequency (or optical) dielectric constant. We have found that the α and γ phases present very similar values of the constant *c* or, equivalently, of ϵ_*∞*_. That results in the close curves for the α and γ phases for Im­[σ] at frequencies above approximately 0.1 eV seen in Figure 7. To be more specific, ignoring some common multiplicative factors, the constant *c* is found to be proportional to 4.1 and 4.4 for α-GeSe in the *x* and *y* directions, and 3.7 for γ-GeSe. The explicit dependence of $\xi (\omega ,d,\vec{k})$ on the values of the constants *c*, in analogy to the case of the effective masses, is in the form of sums and differences of the constants *c* along each direction. Based on these values of the *c*s, the difference in the total heat flux should be limited to approximately 15%. This is consistent with the maximum differences in *Q* between the two phases calculated at short separations, which, albeit larger than those for large separations, are still below 10%.

Thus, even though the optical conductivity of the α- and γ-GeSe differ in so many aspects, as seen in Figures 3 and 7, the fact that the effective electron masses and ϵ_*∞*_ are not too different and the particular dependence of $\xi (\omega ,d,\vec{k})$ on the combinations of these physical parameters result in a very similar overall performance for the α and γ phases.

The similarities between the characteristics of the total heat transfer delivered by the α and γ phases and the difference to the β phase can be made clearer by analyzing the spectral heat flux *q*(ω). In Figure 8a,b, it is possible to see that the differences between *q* for the α and β phases decrease in going from *d* = 10 to 50 nm. At the larger separation, the spectral heat flux is very similar for the two phases. However, at 10 nm, the spectral heat flux of phase α is impacted by its anisotropy as *n*_e_ increases. The anisotropy is reflected in the departure of the curve for *q* from a more bell-shaped format for the larger ω. This effect of the anisotropy becomes dominant for the β phase, as can be seen in Figure 8c,d. The anisotropy broadens the spectral heat flux, as expected; however, the amplitude is diminished correspondingly. In the end, the area contained below the curves (the total flux *Q*) for the isotropic and anisotropic phases has similar values. This decrease in the amplitude of *q* that goes with its spectral broadening frustrates the expected enhancement of the heat flux for the highly anisotropic and hyperbolic β phase.

**Figure 8:**
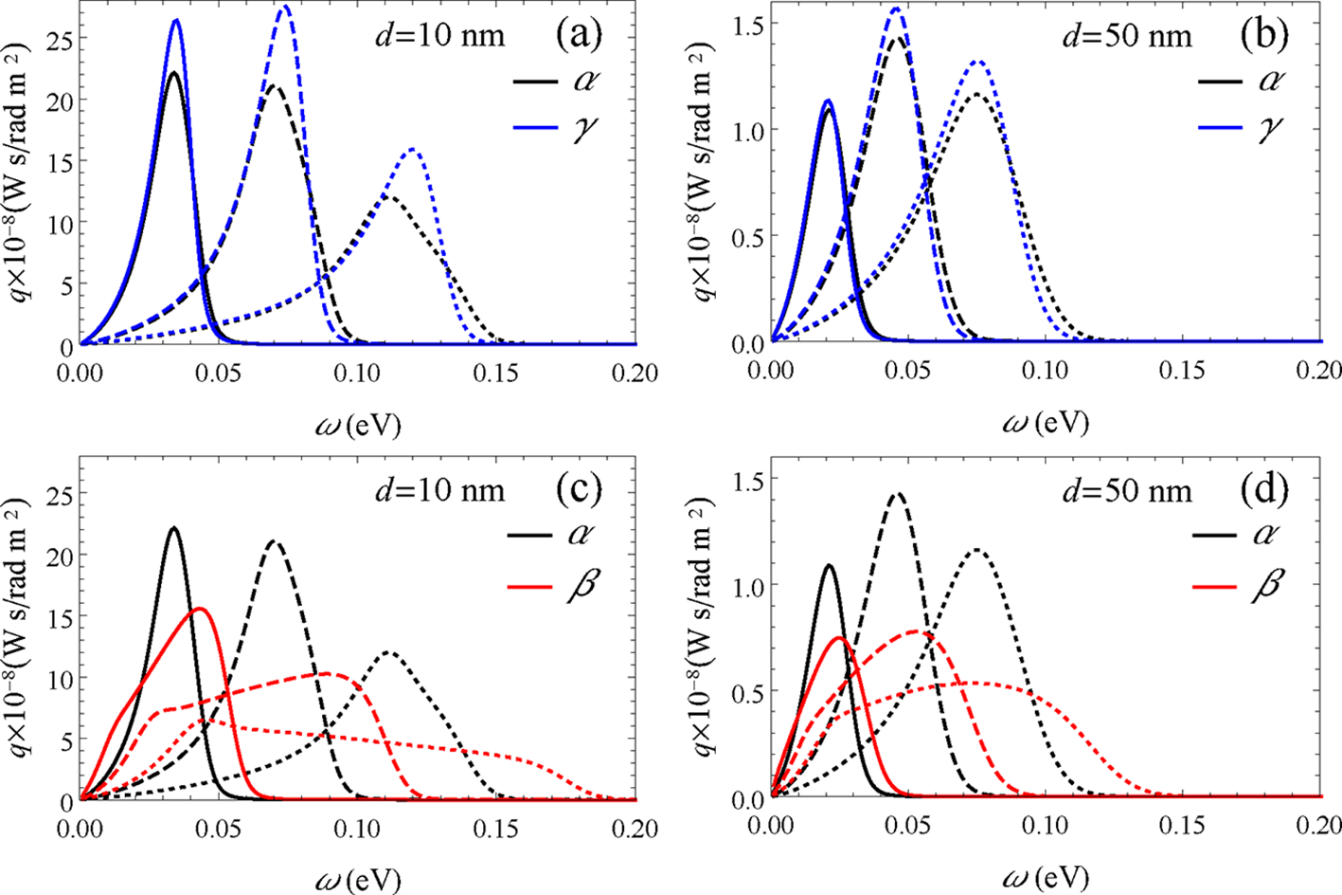
Spectral heat flux *q*(ω) calculated for γ_e_ = 0.01 eV and *n*_e_ = 0.5, 2, and 5 × 10^12^ cm^–2^ corresponding, respectively, to the continuous, dashed, and dotted lines. In (a) and (b) *d* = 10 nm, and in (b) and (d) *d* = 50 nm. In the upper panels, the α and γ phases of GeSe are compared, while in the lower panels the α and β phases are shown.

The fact that the hyperbolic phase does not deliver a maximum heat flux larger than the other nonhyperbolic phases is not absolutely unexpected. While a hyperbolic material has the potential to deliver a larger heat flux, some conditions must be fulfilled for this to happen. In particular, the optical losses can prevent the large *k*_||_ allowed by the hyperbolicity may exist,^56,57^ and the frequency-dependent dielectric function is key to determining the range of frequencies with hyperbolic modes and their transmissivity.^56^ This is well illustrated for the hyperbolic metamaterial made from SiC nanorods considered by Biehs et al.,^56^ which delivered only a slightly larger heat flux compared to that between two homogeneous SiC slabs at separations below 100 nm, and which were significantly smaller beyond that.

In Figure 9, we plot the photon transmission coefficient as a function of the in-plane wave vectors *k*_*x*_ and *k*_*y*_ for the γ and β phases. These plots show which modes contribute significantly to the heat flux. They were plotted for a frequency (ω = 0.07 eV) close to the maximum of the spectral heat flux for the γ phase when *d* = 10 nm and *n*_e_ = 2 × 10^12^ cm^–2^ (see Figure 8a). The spectral heat flux is also high for the β phase under the same conditions (see Figure 8c). What Figure 9 reveals is that the maximum *k*_||_ is similar for both phases, instead of being significantly large for the β phase. Therefore, it is this restriction on *k*_||_ resulting from the particular optical properties of the β phase that prevents an enhanced heat flux compared to those of the other phases. Anyhow, the attained total heat fluxes are quite large for all phases.

**Figure 9:**
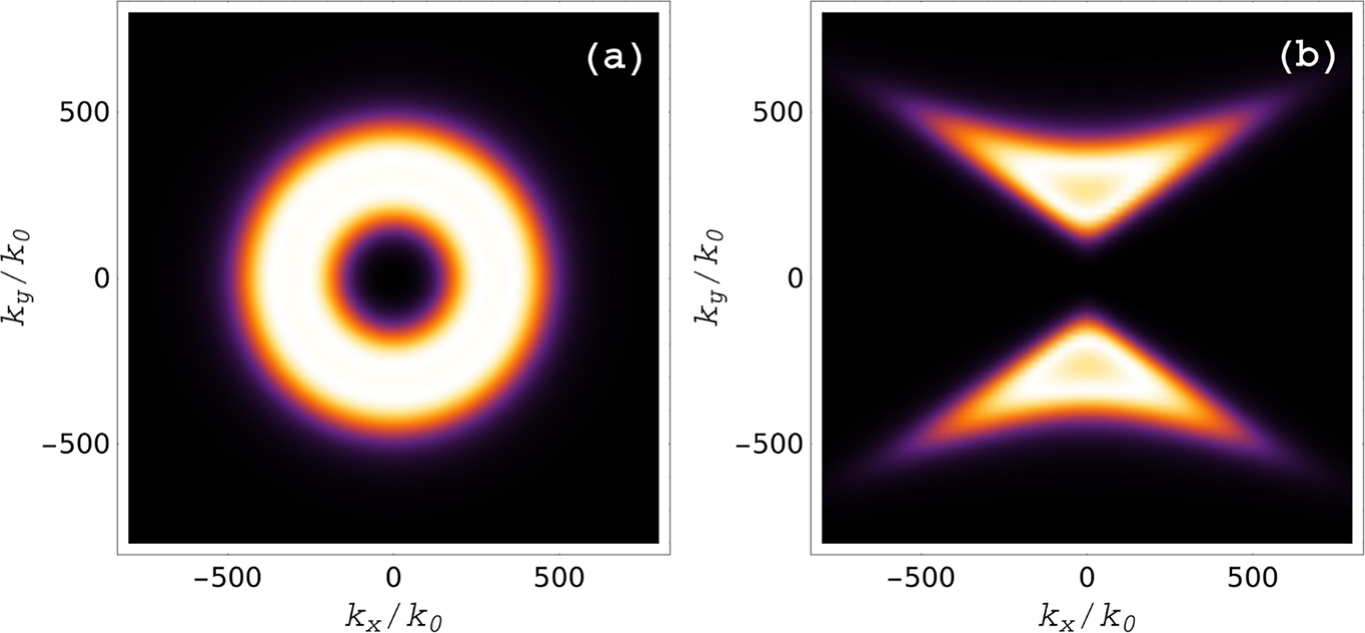
Photon transmission coefficient $\xi (\omega ,d,\vec{k})$ for ω = 0.07 eV and *d* = 10 nm as a funtion of the surface components of the wave vector, *k*_*x*_ and *k*_*y*_ normalized to *k*_0_. The bright colors indicate a transmission closer to 1. Results for (a) the γ phase and (b) the β phase, showing that the maximum values of *k*_*x*_ and *k*_*y*_ are similar for both phases.

## Conclusions

In this work, we have compared the performance of three phases of n-doped monolayer GeSe for application in NFRHT. For that purpose, the electronic and optical properties were obtained from first-principles calculations. The relevant physical properties were the effective electron masses and the intensity of the interband transitions. The electron masses help to set the plasma frequency and the dominant optical response at frequencies below ∼0.1 eV. The low frequency response to the interband transitions around approximately 0.1 eV and above dominates over the contribution of the free electron plasma, as evidenced in our previous results^33^ and Figure 7. The contribution of the optical phonons was ignored based on our previous results for the β phase.^33,53^ Their contribution are expected to alter *Q* significantly only for low doping levels, below *n*_e_ ∼ 0.5 × 10^12^ cm^–2^. Because the maximum *Q* was expected to be at significantly larger values of *n*_e_, as confirmed by our present results, we considered only values of *n*_e_ for which the contribution of the optical phonons was expected to be negligible, that is, *n*_e_ > 0.5 × 10^12^ cm^–2^.

The more important conclusion is that despite the differences in several physical properties of the three phases that affect their optical response, they can deliver similar maximum heat fluxes. Therefore, the choice of which phase is more suitable for practical applications can be influenced by other criteria. For instance, one can choose to use the phase that can be more easily obtained. Some other relevant physical properties of each phase could also be considered to formulate a criterion to choose a particular phase. One such relevant property is thermal conductivity. It determines how efficiently a monolayer can take the absorbed heat away from the heat exchange region to a suitable heat reservoir at a low temperature. Interestingly, the three phases of GeSe have been predicted to have approximately the same lattice thermal conductivity.^58^ Therefore, this property alone does not help to determine the best phase for use in NFRHT. However, as the predicted lattice thermal conductivities are comparatively small (∼4–6 W/m K), the contribution of the electronic thermal conductivity due to the free electrons added by the doping can be relevant. A recent analysis for γ-GeSe^59^ has shown that while the electronic thermal conductivity is small for electron densities ∼10^12^ cm^–2^, it can rapidly increase with *n*_e_ reaching a value close to 4 W/m K for *n*_e_ = 10^13^ cm^–2^. Therefore, if the electronic contribution to thermal conductivity is approximately the same for all phases, then it is going to be small for the electron densities that maximize *Q* for α- and γ-GeSe. In its turn, for the β phase it can be significant due to the maximization of *Q* at *n*_e_ close to or higher than 10^13^ cm^–2^. Therefore, for freestanding monolayers, the use of β-GeSe is favored. However, if the GeSe monolayers are used on top of good thermal conductors, their low thermal conductivity is not an issue, and the more appropriate phase can be chosen based on some other aspect.

It was interesting to conclude that the performance of the highly anisotropic β phase does not surpass that of the other two phases. As discussed by the authors in a previous work,^53^ β-GeSe exhibits hyperbolicity over a large range of frequencies. The α phase can also be hyperbolic; however, only in a very narrow frequency range, whose effects are negligible. It is usually expected that hyperbolic materials can deliver larger heat fluxes compared to isotropic or nonhyperbolic anisotropic materials.^42,56^ This frustration with the performance of monolayer β-GeSe was also found when it was compared with multilayer β-GeSe,^53^ which is less anisotropic and presents smaller ranges of hyperbolicity. As the anisotropy does not stand as a necessary property of a 2D material to deliver large heat fluxes, it is worth further investigating which physical properties are more relevant, particularly when doping is involved.

The analyses presented in this work contribute to a better understanding of the electronic and optical properties of experimentally relevant phases of monolayer GeSe, a novel and interesting 2D material, through an ab initio approach. The calculation of the optical properties based on consistent DFT calculations for the three phases has allowed an adequate comparison of their performance in application to NFRHT. The fact that the different phases can deliver similar maximum heat fluxes also raises the question of whether this is a particular property of GeSe or whether different phases of other 2D materials can be expected to behave similarly.

## References

[1] Biehs S.-A., Ben-Abdallah P., Rosa F. S., Joulain K., Greffet J.-J. (2011). Nanoscale
heat flux between nanoporous materials. Opt.
Express.

[2] Volokitin, A. I. ; Persson, B. N. Electromagnetic Fluctuations at the Nanoscale: Theory and Applications, 1st ed.; Springer: Heidelberg, 2017.

[3] Kittel A., Müller-Hirsch W., Parisi J., Biehs S.-A., Reddig D., Holthaus M. (2005). Near-Field Heat Transfer in a Scanning Thermal Microscope. Phys. Rev. Lett..

[4] De
Wilde Y., Formanek F., Carminati R., Gralak B., Lemoine P.-A., Joulain K., Mulet J.-P., Chen Y., Greffet J.-J. (2006). Thermal radiation scanning tunelling
microscopy. Nature.

[5] Francoeur M. (2015). Near-field
radiative energy transfer: Nanostructures feel the heat. Nat. Nanotechnol..

[6] Rousseau E., Siria A., Jourdan G., Volz S., Comin F., Chevrier J., Greffet J.-J. (2009). Radiative heat transfer at the nanoscale. Nat. Photonics.

[7] Hargreaves C. (1969). Anomalous
radiative transfer between closely-spaced bodies. Phys. Lett. A.

[8] Polder D., Van Hove M. (1971). Theory of Radiative Heat Transfer
between Closely Spaced
Bodies. Phys. Rev. B.

[9] Vinogradov E. A., Dorofeev I. A. (2009). Thermally stimulated
electromagnetic fields of solids. Phys.-Uspekhi.

[10] Ben-Abdallah P., Biehs S.-A. (2014). Near-Field Thermal
Transistor. Phys. Rev. Lett..

[11] Ben-Abdallah P., Biehs S.-A. (2017). Thermotronics: Towards
Nanocircuits to Manage Radiative
Heat Flux. Z. Naturforsch. A.

[12] Shi K., Sun Y., Chen Z., He N., Bao F., Evans J., He S. (2019). Colossal enhancement
of near-field thermal radiation across hundreds
of nanometers between millimeter-scale plates through surface plasmon
and phonon polaritons coupling. Nano Lett..

[13] Pérez-Rodríguez J. E., Pirruccio G., Esquivel-Sirvent R. (2017). Fano interference for tailoring near-field
radiative heat transfer. Phys. Rev. Mater..

[14] Esquivel-Sirvent R. (2016). Ultra thin
metallic coatings to control near field radiative heat transfer. AIP Adv..

[15] Pérez-Rodríguez J. E., Pirruccio G., Esquivel-Sirvent R. (2019). Spectral gaps in the near-field heat
flux. Phys. Rev. Materials.

[16] Castillo-López S., Pirruccio G., Villarreal C., Esquivel-Sirvent R. (2020). Near-field
radiative heat transfer between high-temperature superconductors. Sci. Rep..

[17] Biehs S.-A., Ben-Abdallah P. (2017). Near-Field
Heat Transfer between Multilayer Hyperbolic
Metamaterials. Z. Naturforsch. A.

[18] Rodriguez-López P., Tse W.-K., Dalvit D. A. (2015). Radiative heat transfer in 2D Dirac
materials. J. Phys.: Cond. Matt..

[19] Zhou C.-L., Wu X.-H., Zhang Y., Yi H.-L., Antezza M. (2021). Polariton
topological transition effects on radiative heat transfer. Phys. Rev. B.

[20] Wu H., Liu X., Cai Y., Cui L., Huang Y. (2022). Near-field
radiative
heat transfer modulated by nontrivial topological surface states. Mater. Today Phys..

[21] Hu Y., Liu H., Yang B., Shi K., Antezza M., Wu X., Sun Y. (2023). High-rectification near-field radiative thermal diode
using Weyl
semimetals. Phys. Rev. Mater..

[22] Fernandez-Hurtado V., Fernandez-Dominguez A. I., Feist J., García-Vidal F.
J., Cuevas J. C. (2018). Exploring
the limits of Super-Planckian far-field radiative
heat transfer using 2D materials. ACS Photonics.

[23] Ge L., Cang Y., Gong K., Zhou L., Yu D., Luo Y. (2018). Control of near-field
radiative heat transfer based on anisotropic
2D materials. AIP Adv..

[24] Othman M. A., Guclu C., Capolino F. (2013). Graphene-based tunable hyperbolic
metamaterials and enhanced near-field absorption. Opt. Express.

[25] Hu Y., Li H., Zhu Y., Yang Y. (2020). Enhanced Near-Field Radiative Heat
Transport between Graphene Metasurfaces with Symmetric Nanopatterns. Phys. Rev. Appl..

[26] Yang F., Song B. (2022). Strong suppression
of near-field thermal transport between twisted
bilayer graphene near the magic angle. Mater.
Today Phys..

[27] He M., Qi H., Ren Y., Zhao Y., Antezza M. (2020). Active control of near-field
radiative heat transfer by a graphene-gratings coating-twisting method. Opt. Lett..

[28] Yi X.-J., Hong X.-J., Shehzad K., Wang T.-B., Xu X.-M., Liao Q.-H., Yu T.-B., Liu N.-H. (2019). Near-field radiative
heat transfer between black phosphorus and graphene sheet. Mater. Res. Express.

[29] Debu D. T., Doha M. H., Churchill H. O., Herzog J. B. (2019). Gate voltage and
doping effects on near-field radiation heat transfer in plasmonic
heterogeneous pairs of graphene and black phosphorene. RSC Adv..

[30] Yang B., Pan D., Guo X., Hu H., Dai Q. (2022). Substrate effects on
the near-field radiative heat transfer between bi-planar graphene/hBN
heterostructures. Int. J. Therm. Sci..

[31] Geim A. K., Grigorieva I. V. (2013). Van der
Waals heterostructures. Nature.

[32] Montblanch A. R.-P., Barbone M., Aharonovich I., Atatüre M., Ferrari A. C. (2023). Layered materials as a platform for
quantum technologies. Nat. Nanotechnol..

[33] Esquivel-Sirvent R., Gusso A., Sánchez
Ochoa F. (2023). Near-Field Radiative
Heat Transfer between β- GeSe monolayers: An ab initio study. Nanoscale and Microscale Thermophys. Eng..

[34] Zhou C.-L., Wu X.-H., Zhang Y., Yi H.-L., Novko D. (2021). Near-field
thermal radiation of germanium selenide single layer. Phys. Rev. Mater..

[35] Liu X., Mao Y. (2024). Promising transport
properties of multifunctional monolayer GeSe
nanodevices. J. Mater. Chem. C.

[36] Xu Y., Xu K., Ma C., Chen Y., Zhang H., Liu Y., Ji Y. (2020). Novel two-dimensional
β-GeSe and β-SnSe semiconductors:
anisotropic high carrier mobility and excellent photocatalytic water
splitting. J. Mater. Chem. A.

[37] Xu Y., Zhang H., Shao H., Ni G., Li J., Lu H., Zhang R., Peng B., Zhu Y., Zhu H., Soukoulis C. M. (2017). First-principles study on the electronic,
optical,
and transport properties of monolayer α- and β-GeSe. Phys. Rev. B.

[38] von
Rohr F. O., Ji H., Cevallos F. A., Gao T., Ong N. P., Cava R. J. (2017). High-Pressure Synthesis and Characterization
of β-GeSe—A Six-Membered-Ring Semiconductor in an Uncommon
Boat Conformation. J. Am. Chem. Soc..

[39] Odebowale A. A., Berhe A., Ogundare R. T., Abdo S., Abdulghani A., Hattori H. T., Miroshnichenko A. E. (2025). Advances
in Radiative Heat Transfer:
Bridging Far-Field Fundamentals and Emerging Near-Field Innovations. Adv. Funct. Mater..

[40] Vinogradov E. A., Dorofeev I. A. (2009). Thermally stimulated
electromagnetic fields of solids. Phys.-Usp..

[41] Liu X., Wang L., Zhang Z. M. (2015). Near-Field
Thermal Radiation: Recent
Progress and Outlook. Nanoscale Microscale Thermophys.
Eng..

[42] Ma L., Le D.-N., Ben-Abdallah P., Woods L. M. (2025). Bulk Layered Materials
and Their Monolayer Counterparts for Radiative Heat Transfer. ACS Appl. Opt. Mater..

[43] Lakhtakia A. (1992). Green’s
functions and Brewster condition for a halfspace bounded by an anisotropic
impedance plane. Int. J. Infrared Millimeter
Waves.

[44] Gomez-Diaz J., Tymchenko M., Alù A. (2015). Hyperbolic metasurfaces: surface
plasmons, light-matter interactions, and physical implementation using
graphene strips. Opt. Mater. Exp..

[45] Joulain K., Mulet J.-P., Marquier F., Carminati R., Greffet J.-J. (2005). Surface electromagnetic waves thermally
excited: Radiative
heat transfer, coherence properties and Casimir forces revisited in
the near field. Surf. Sci. Rep..

[46] Kresse G., Furthmüller J. (1996). Efficient
iterative schemes for ab initio total-energy
calculations using a plane-wave basis set. Phys.
Rev. B.

[47] Kresse G., Joubert D. (1999). From ultrasoft pseudopotentials
to the projector augmented-wave
method. Phys. Rev. B.

[48] Guan S., Liu C., Lu Y., Yao Y., Yang S. A. (2018). Tunable ferroelectricity
and anisotropic electric transport in monolayer β-GeSe. Phys. Rev. B.

[49] Luo N., Wang C., Jiang Z., Xu Y., Zou X., Duan W. (2018). Saddle-point excitons and their extraordinary light absorption in
2D β-phase group-IV monochalcogenides. Adv. Funct. Mater..

[50] Zhou J., Zhang S. (2021). Terahertz optics-driven
phase transition in two-dimensional multiferroics. npj 2D Mater. Appl..

[51] Perdew J. P., Burke K., Ernzerhof M. (1996). Generalized
gradient approximation
made simple. Phys. Rev. Lett..

[52] Csonka G. I., Perdew J. P., Ruzsinszky A., Philipsen P. H., Lebègue S., Paier J., Vydrov O. A., Ángyán J. G. (2009). Assessing
the performance of recent density functionals for bulk solids. Phys. Rev. B.

[53] Gusso A., Sánchez-Ochoa F., Esquivel-Sirvent R. (2024). Near-Field
Radiative Heat Transfer
between Layered β-GeSe Slabs: First-Principles Approach. Langmuir.

[54] Monkhorst H. J., Pack J. D. (1976). Special points for Brillouin-zone
integrations. Phys. Rev. B.

[55] Lee S., Jung J.-E., Kim H.-G., Lee Y., Park J. M., Jang J., Yoon S., Ghosh A., Kim M., Kim J. (2021). γ-GeSe: a new hexagonal polymorph from
group
IV–VI monochalcogenides. Nano Lett..

[56] Biehs S.-A., Tschikin M., Ben-Abdallah P. (2012). Hyperbolic
Metamaterials as an Analog
of a Blackbody in the Near Field. Phys. Rev.
Lett..

[57] Biehs S.-A., Rousseau E., Greffet J.-J. (2010). Mesoscopic
Description of Radiative
Heat Transfer at the Nanoscale. Phys. Rev. Lett..

[58] Wang B., Yan X., Cui X., Cai Y. (2022). First-Principles Study of the Phonon
Lifetime and Low Lattice Thermal Conductivity of Monolayer γ-GeSe:
A Comparative Study. ACS Appl. Nano Mater..

[59] Shu Z., Wang B., Cui X., Yan X., Yan H., Jia H., Cai Y. (2023). High-performance thermoelectric
monolayer γ-GeSe
and its group-IV monochalcogenide isostructural family. Chem. Eng. J..

